# Morphological and molecular characterization of *Alternaria alternata* causing Alternaria blight in lentil (*Lens culinaris* Medik.) and evaluation of the disease reactions of selected lentil cultivars in Türkiye

**DOI:** 10.7717/peerj.21834

**Published:** 2026-11-09

**Authors:** Nisa Ertoy

**Affiliations:** Vocational High School of Technical Sciences Department of Plant and Animal Production, Akdeniz University, Antalya, Turkey

**Keywords:** *Lens culinaris*, *Alternaria alternata*, Disease severity, Host resistance, Pathogenicity

## Abstract

Lentil (*Lens culinaris* Medik.) is one of the most important edible legumes grown in Türkiye. However, production is increasingly threatened by leaf blight, and *Alternaria alternata* (Fries) Keissler has emerged as an important causal agent of Alternaria blight. This study aimed to determine the pathogenicity of three *A. alternata* isolates (NELeAa-1, NELeAa-2, and NELeAa-3) collected between 2023 and 2024 from major lentil-producing provinces in Türkiye, and to evaluate the resistance of eight common lentil varieties (four winter and four summer types). Isolates were identified based on morphological characteristics and dual-locus molecular analyses. To ensure accurate taxonomic identification, in addition to standard Internal Transcribed Spacer (ITS) rDNA sequencing, the species-specific AaltFor/AaltRev primer set was used to target the beta-tubulin (*β*-tubulin) gene region. Two-way Analysis of Variance (ANOVA) showed that disease severity was significantly influenced by both the fungal isolate and the host variety (*P* < 0.001), with a strong interaction between pathogen virulence and host resistance. Disease severity scores ranged from 15.33% to 71.00%, demonstrating significant variation among cultivars. While the summer varieties ‘Meyveci 2001’ and ‘Karagül’ were the most susceptible (S), winter varieties such as ‘Çiftçi’ and ‘Ceren’ showed a stronger moderately resistant (MR) profile. Isolate NELeAa-2 had the highest virulence, particularly on summer cultivars, whereas NELeAa-1 was more virulent on certain winter-type cultivars. Our findings suggest that utilizing the genetic resistance of winter varieties provides a viable breeding strategy to mitigate Alternaria blight under changing climate conditions.

## Introduction

Lentil (*Lens culinaris* Medik.) is a globally significant legume, valued in human nutrition as a rich source of plant-based protein, essential minerals, and dietary fiber (FAOSTAT, 2024; Sarker & Erskine, 2022). Widely cultivated in semi-arid regions such as Türkiye, this crop contributes to sustainable agricultural systems through its ability to fix atmospheric nitrogen and adapt to marginal environmental conditions (Rawal & Navarro, 2019). Currently, lentils are grown in over 40 countries, with a total global production of 7,887,380 tonnes. Although the crop’s center of origin is the ‘Fertile Crescent’, a region encompassing Southeastern Anatolia, Northern Syria, and Mesopotamia (Zohary, Hopf & Weiss, 2012), Canada currently leads global production. Türkiye, with a production of 405,000 tonnes, is a major producer and the world’s third-largest importer after India and Bangladesh (FAOSTAT, 2024). This highlights Türkiye’s role, which serves as an important global lentil processing hub. According to 2024 data, lentil remains the second most produced food legume in Türkiye after chickpeas (TUIK, 2025).

Despite its importance in domestic agriculture and Türkiye’s position in the global trade, production is often limited by various biotic stresses, particularly fungal diseases that affect both yield and quality. Among these, Alternaria blight, caused by *Alternaria alternata* (Fries) Keissler, is increasingly recognized as an economically significant pathogen in many lentil-growing regions (Bashir & Ahmad, 2020; Meena et al., 2017). This pathogen causes necrotic lesions on the aerial parts of the plant, leading to defoliation, reduced photosynthetic capacity, and ultimately, substantial yield losses (Bashir & Ahmad, 2020).

The *Alternaria* genus includes more than 300 species worldwide, infecting a wide range of cultivated plants and leading to significant economic damage (Lawrence et al., 2016). In Türkiye, Alternaria species have been identified across various regions, causing substantial losses in economically important crops such as tomato, potato, bean, and various fruit trees (Kaya & Zorba, 2021). *Alternaria alternata* is a widely distributed fungal pathogen with a broad host range and high ecological adaptability. Its ability to produce airborne conidia allows rapid dispersal under favorable environmental conditions, especially in regions with high humidity and moderate temperatures (Lawrence et al., 2016). Recent studies report an increasing frequency and severity of Alternaria blight in legume crops, suggesting that the pathogen has become a more serious threat under changing climatic conditions (Bashir & Ahmad, 2020; Ertoy, 2022). Despite the rising disease pressure, the taxonomic identification of *Alternaria* species remains challenging due to morphological similarities within the small-spored species complex.

Accurate identification of fungal pathogens is essential for developing effective disease management strategies. While morphological characterization is a fundamental approach, it is often insufficient because of variability among isolates and similarities between closely related species (Lawrence et al., 2016). Therefore, molecular tools are necessary for precise identification. Specifically, sequencing of the internal transcribed spacer (ITS) region and protein-coding genes such as *β*-tubulin is widely used for the reliable identification and phylogenetic analysis of fungal species (Schoch et al., 2012; Woudenberg et al., 2015).

Beyond pathogen identification, evaluating host plant responses is important for developing sustainable breeding strategies. Evaluating disease reactions of cultivars is one of the most effective and environmentally friendly ways to manage the disease (Poland et al., 2009; Sarker & Erskine, 2022). However, information regarding the resistance levels of lentil cultivars grown in Türkiye against *A. alternata* is still limited.

Therefore, this study was designed with two primary objectives: (i) to perform morphological and molecular characterization of *Alternaria alternata* isolates obtained from major lentil-producing provinces in Türkiye (Ankara, Konya, and Yozgat), and (ii) to evaluate the disease responses of summer and winter lentil cultivars under controlled conditions. The findings aim to provide a scientific basis for regional breeding programs and integrated disease management strategies.

## Materials and Methods

### Isolation and morphological identification of the pathogen

Fungal isolates were obtained from symptomatic lentil leaves showing characteristic blight lesions. Leaf samples were collected from Ankara, Konya, and Yozgat provinces, the major lentil-producing regions in Türkiye, during the 2023–2024 growing seasons. Sampling and disease assessments were conducted in accordance with the national technical instructions for integrated pest management in lentils (Ministry of Food, Agriculture and Livestock, 2011). Following the protocol described by Kouadri, Bekkar & Zaim (2023a), diseased leaf tissues were surface-sterilized with 1% sodium hypochlorite (NaOCl) for 3 min, rinsed three times with sterile distilled water, and placed on Potato Dextrose Agar (PDA). Plates were incubated at 25 ± 1 °C for 7 days in the dark. Pure cultures were obtained using the single-spore isolation technique. Isolates were identified based on colony morphology and conidial characteristics using the taxonomic criteria and standard cultural conditions described by Simmons (2007).

### DNA extraction and polymerase chain reaction

The three fungal isolates (NELeAa-1, NELeAa-2, and NELeAa-3) used for the polymerase chain reaction (PCR) reactions were cultured on PDA and incubated at 25 °C for 7–10 days. Genomic DNA was extracted from fungal mycelia and conidia using the CTAB extraction protocol (Doyle, 1990) with minor modifications. The quality and quantity of the extracted genomic DNA were assessed using a NanoDrop™ 2000 spectrophotometer (Thermo Fisher Scientific, Wilmington, DE, USA), and the DNA concentration was adjusted to approximately 50 ng/µL for subsequent PCR amplifications. Molecular identification was performed using ITS1/ITS4 primers for the ITS region (White et al., 1990). Additionally, AaltFor/AaltRev primers specific to the *β*-tubulin gene region were used for species-level confirmation (Kordalewska et al., 2015).

The amplification reactions were carried out in a total volume of 25 µL containing 2.5 µL of 10× PCR buffer, 0.5 µL of dNTP mix (10 mM each), 1 µL of each forward and reverse primer (10 µM), 0.2 µL of Taq DNA Polymerase (5 U/µL; Thermo Fisher Scientific, Wilmington, DE, USA), 1.5 µL of genomic DNA (∼50 ng), and sterile nuclease-free water to complete the final volume. PCR amplifications were executed using a T100™ Thermal Cycler (Bio-Rad Laboratories, Hercules, CA, USA).

The general thermal cycling conditions consisted of an initial denaturation step at 95 °C for 5 min, followed by 35 cycles of denaturation at 94 °C for 30 s, annealing at 55 °C for 30 s, and extension at 72 °C for 1 min, with a final extension step at 72 °C for 10 min. The specific primer sequences and optimized internal thermal protocols for each gene region are summarized in Table 1.

**Table 1:** Primer pairs and PCR protocols used in this study.

**Primers and references**	**Sequence**	**PCR conditions**		
		Temperature (°C)	Time (seconds)	Cycles
ITS1/ ITS4 White et al. (1990)	5′-TCCGTAGGTGAACCTGCGG-3′ 5′-TCCTCCGCTTATTGATATGC-3′			
		94	300	1
		94	30	30
		56	60	
		72	90	
		72	300	1
AaltFor/ AaltRev Kordalewska et al. (2015)	5′-GTGCCTTCCCCCAAGGTCTCCG-3′ 5′-CGGAAACGAGGTGGTTCAGGTC-3′	95	300	1
		95	60	35
		58	60	
		72	60	
		72	300	1

### DNA sequencing and phylogenetic analysis

The PCR products from the ITS region of the three isolates were sequenced by Macrogen Inc. (Amsterdam, Netherlands). Sequence editing and multiple sequence alignment were performed using the ClustalW algorithm in BioEdit (v7.2.5) software (Hall, 1999). Sequences were compared with the NCBI GenBank database using BLASTn (Altschul et al., 1990). While the *β*-tubulin sequences were used for species confirmation, phylogenetic analysis was based on the ITS rDNA dataset. All sequences were deposited in GenBank. Phylogenetic trees were constructed using the Maximum Likelihood (ML) method based on the Tamura 3-parameter model in MEGA 5.1 (Tamura et al., 2011). Reliability was evaluated using bootstrap analysis with 1,000 replicates.

### Plant material and experimental conditions

A total of eight green lentil (*Lens culinaris* Medik.) cultivars, including four winter cultivars (Ankarayeşili, Ceren, Çiftçi, and Yusufhan) and four summer cultivars (‘Bozok’, ‘Gümrah’, ‘Karagül’, and ‘Meyveci 2001’) were used in this study. All cultivars were officially registered in Türkiye and were obtained from the Central Research Institute for Field Crops (CRIFC), Ankara. For the pathogenicity assays, three seeds of each cultivar were sown in three kg plastic pots containing a sterile mixture of peat and perlite (1:1, v/v). For each specific cultivar–isolate combination, a total of three independent pots were deployed as replicates, with each individual pot hosting three plants. All three representative isolates (NELeAa-1, NELeAa-2, and NELeAa-3) were comprehensively tested across all eight cultivars in this trial. The experiments were conducted in November 2025 in a research greenhouse at Akdeniz University (Antalya, Türkiye). Environmental conditions were maintained to support Alternaria blight development. The greenhouse temperature was regulated at 25 ± 2 °C with a relative humidity profile of 70%–80%, under a controlled 12-hour light/12-hour dark photoperiod cycle. The experiment was conducted using a completely randomized design (CRD) with three replicates for each cultivar–isolate combination.

### Inoculation and disease assessment

Conidial inoculum was prepared from 10-day-old cultures grown on PDA, and the final concentration of the suspension was adjusted to 1 × 10^5^ conidia/mL using a Neubauer hemocytometer. To ensure uniform spore dispersion, Tween 20 (0.05%, v/v) was added to the suspension. Plants were inoculated using a handheld foliar spray method until run-off. Each pot was covered with a clear polyethylene bag for 24 h after inoculation to maintain a high relative humidity level (approximately 95%–100%) required for successful infection. Plants were monitored daily, and disease severity was evaluated 14 days post-inoculation (dpi). Disease severity was determined by visually estimating the average percentage of infected leaf area evaluated across all leaves per plant within each pot, as described by Bock & Chiang (2022). Cultivar reactions were categorized as Immune (I, 0%), Resistant (R, 1–10%), Moderately Resistant (MR, 11–25%), Moderately Susceptible (MS, 26–50%), Susceptible (S, 51–75%), or Highly Susceptible (HS, 76–100%) as shown in Table 2.

**Table 2:** The 0–9 scale and infected area percentages used for the classification of phenotypic reaction categories of lentil cultivars against *Alternaria* blight. Disease rating scale (0–9) and phenotypic reaction categories used for the evaluation of *Alternaria* blight on lentil cultivars.

**Scale value**	**Infected area (%)**	**Reaction code**	**Phenotypic description**
0	%0	I	Immune
1	%1–10	R	Resistant
3	%11–25	MR	Moderately resistant
5	%26–50	MS	Moderately susceptible
7	%51–75	S	susceptible
9	%76–100	HS	Highly susceptible

### Statistical analysis

To evaluate the effects of isolates, cultivars, and their interaction on disease severity, data were subjected to a two-way Analysis of Variance (ANOVA). Normality and homogeneity of variances were verified before analysis. Mean separations were performed using Duncan’s multiple range test (DMRT) at *P* < 0.05. Statistical computations were performed using R software (v4.3.1) with the ‘agricolae’ package (Mendiburu, 2021).

## Results

### Recovery and morphological identification of isolates

Lentil leaves showing symptoms of Alternaria blight were collected from fields in Ankara, Konya, and Yozgat provinces, which are major green lentil-producing regions in Türkiye. Following the isolation procedures, three representative fungal isolates, NELeAa-1 (Ankara), NELeAa-2 (Konya), and NELeAa-3 (Yozgat), were recovered from the symptomatic tissues. All isolates showed rapid radial growth on PDA at 25 °C, completely covering the 90 mm petri dishes within 7 days. Morphological observations aligned with the taxonomic descriptions of *Alternaria alternata*; colonies initially appeared light gray and became dark olive-green to velvet-black with age, featuring a dense aerial mycelium (Fig. 1).

**Figure 1:**
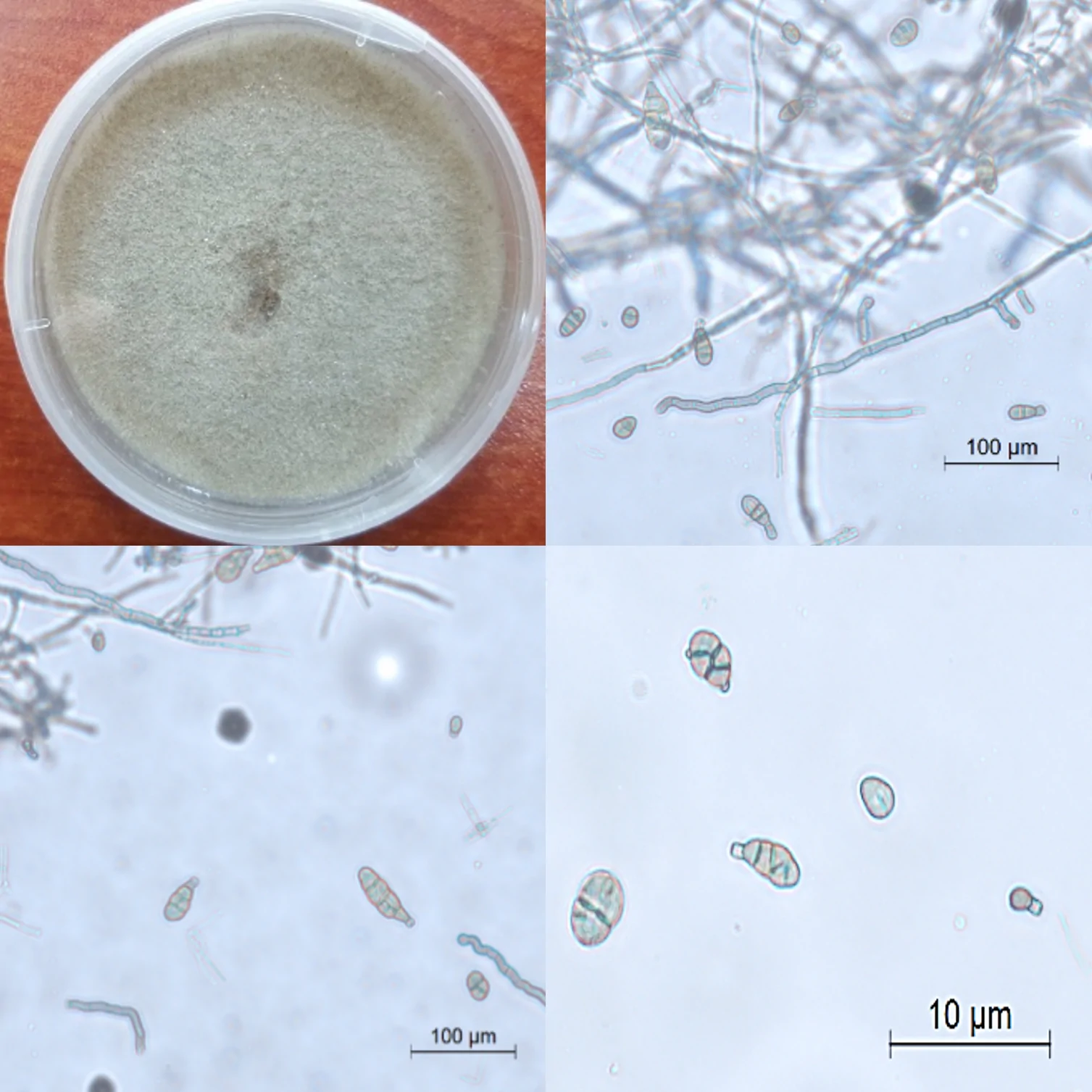
Morphological characteristics of *Alternaria alternata*. (A) Growth in PDA medium; (B, C, D) the view under the microscope (40×).

Microscopic analysis under a light microscope at 400× revealed the presence of typical multiseptate conidia, and a total of 50 conidia were measured for each representative isolate to determine morphological dimensions. The conidia were primarily light to dark brown, obclavate to piriform in shape, and arranged in short chains. Conidial arrangement on PDA showed varying chain lengths depending on the plant tissue: 4–9 conidia on leaves, 3–8 on pods, and 2–7 on stems. Across all substrates, the conidia were characterized by tapering apical beaks and prominent primary septa. These spores were typically ellipsoidal or ovoid, featuring cylindrical, short beaks and elongated, thin septa. The mean conidial size for the isolates was 18.6 × 14.2 µm on PDA. Both longitudinal and transverse septa were clearly visible (Fig. 1). These characteristics were consistent with the descriptions of *Alternaria alternata* and were subsequently confirmed by molecular analysis.

### Molecular characterization and phylogenetic analysis

The ITS region was amplified using the universal primer pairs ITS1/ITS4 (White et al., 1990), yielding products of approximately 560 bp. The species-specific primer pairs Alt-for/Alt-rev (Kordalewska et al., 2015) were used to confirm identifications, producing the expected 184 bp amplicons. These molecular results supported the initial morphological identification, identifying the isolates as *A. alternata*.

**Table 3:** Accession numbers of *Alternaria spp*. isolates obtained from GenBank used in construction of phylogenetic treesGenBank nucleotide accessions of tested *Alternaria alternata* isolates along with other *Alternaria* species based on ITS region used for phylogenetic analysis.

**Species**	**Isolate name**	**Accession no.**	**Species**	**Isolate name**	**Accession no.**
*Alternaria alternata*	NELeAa-1	PZ231509	*Alternaria alternata*	WJQ	OM319520
*Alternaria alternata*	NELeAa-2	PZ231510	*Alternaria alternata*	GFRS07	MT487764
*Alternaria alternata*	NELeAa-3	PZ231511	*Alternaria chlamydosporigena*	LYZ1302	PV926706
*Alternaria alternata*	HC-2	MT644140	*Alternaria chlamydosporigena*	LYZ1299	PV926703
*Alternaria alternata*	ZSY-2-1	MT487774	*Alternaria chlamydosporigena*	ZH-45	PV019415
*Alternaria alternata*	LaA3	MN989198	*Alternaria dauci*	CBS 111.38	NR136087
*Alternaria alternata*	BJFA-1	MK886520	*Alternaria dauci*	18S	EU781950
*Alternaria alternata*	BJFA-3	MK886521	*Alternaria dauci*	US-FL-1 18S	MF595073
*Alternaria alternata*	4sp1	MN525798	*Alternaria dauci*	BM-2	MZ361739
*Alternaria alternata*	PF-038	MN752162	*Alternaria tenuissima*	psfsd9	MN853397
*Alternaria alternata*	Aa-6	PV750660	*Alternaria tenuissima*	43b 1	MT362612
*Alternaria alternata*	ss-3	PP499318	*Alternaria tenuissima*	N10L3	MT134981
*Alternaria alternata*	KSPM 135	PX457273	*Alternaria tenuissima*	ZP4-3	MT487771

Phylogenetic relationships were analyzed by aligning the ITS sequences of the isolates with reference sequences from the NCBI GenBank database (Table 3) using MEGA 5.1 software. A Maximum Likelihood tree was constructed based on the Tamura 3-parameter model with 1,000 bootstrap replicates (Tamura, 1992; Tamura et al., 2011). The phylogenetic analysis showed that the morphologically identified isolates clustered within the same clade as the *A. alternata* reference isolates from GenBank (Fig. 2). The isolates NELeAa-1, NELeAa-2, and NELeAa-3 grouped with GenBank accessions HC-2, ZSY-2-1, LaA3, BJFA-1, BJFA-3, 4sp1, PF-038, Aa-6, ss-3, KSPM 135, WJQ, and GFRS-07, with a BLASTn sequence similarity of 99%. This group was clearly distinct from other species such as *A. tenuissima*, *A. dauci*, and *A. chlamydosporigena*, which formed separate clades.

**Figure 2:**
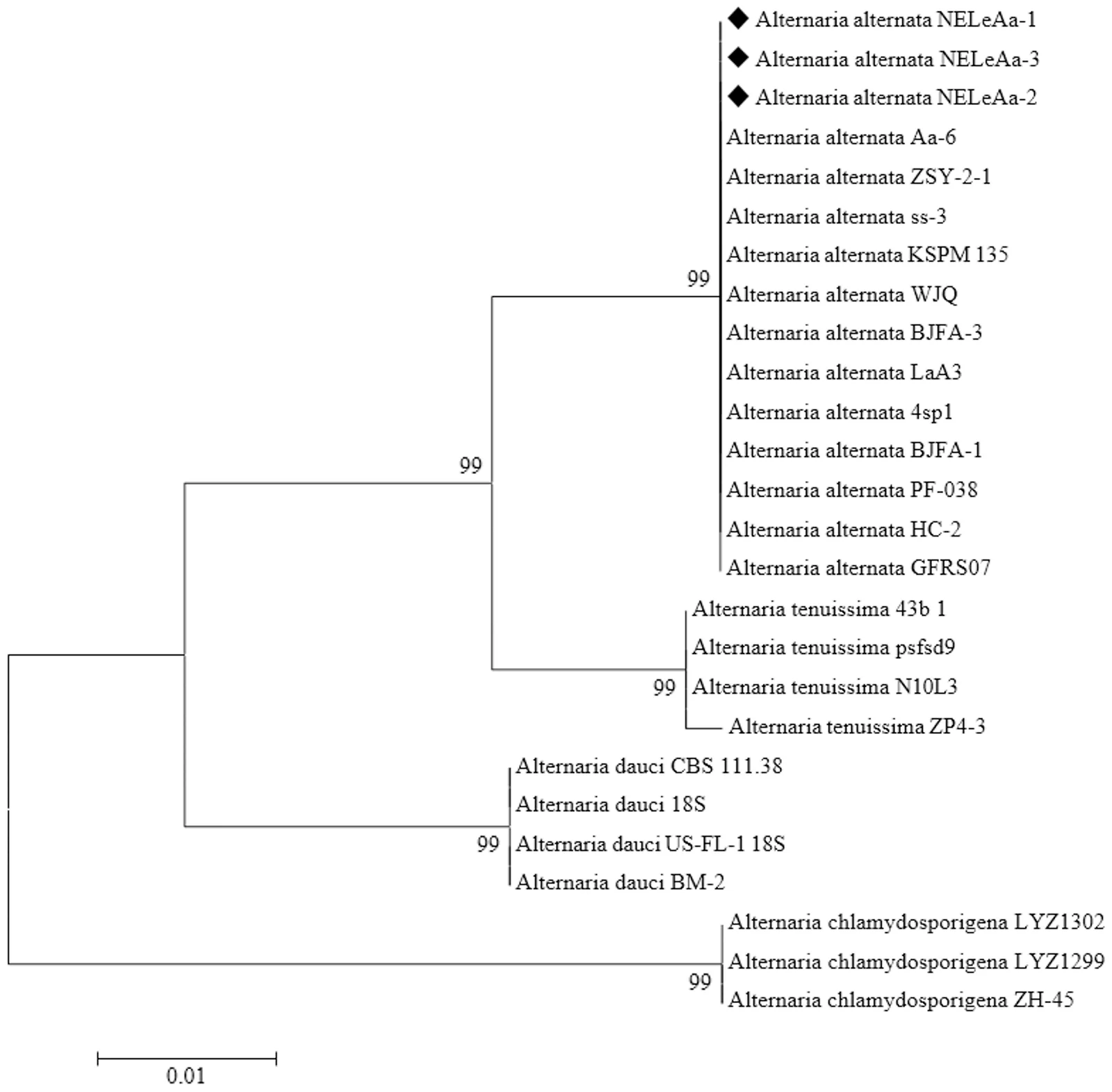
Molecular phylogenetic analysis of *Alternaria alternata* isolates of ITS gene region. Dendrogram showing genetic relatedness of Alternaria alternata (three isolates) isolated from leaves of lentil planted in Türkiye, compared against sequences of *A. alternata* (12 isolates), *A. tenuissima* (four isolates), *A. dauci* (four isolates) and *A. chlamydosporigena* (three isolates) from GenBank (Table 3) (= shows isolates obtained in this study).

### Pathogenicity assays and host resistance

The pathogenicity of all isolates was confirmed using the foliar spray inoculation method. Initial symptoms appeared as small, chlorotic pin-point lesions on leaves 4–5 days after inoculation. By 14 days, these lesions had developed into characteristic “Alternaria blight” symptoms: irregular, dark brown necrotic spots often surrounded by a chlorotic halo. In highly susceptible cultivars, these lesions coalesced, leading to extensive leaf blighting and premature defoliation (Figs. 3 and 4). No symptoms were observed on the control plants sprayed with sterile distilled water, confirming that the disease was due to the inoculated *A. alternata* isolates.

**Figure 3:**
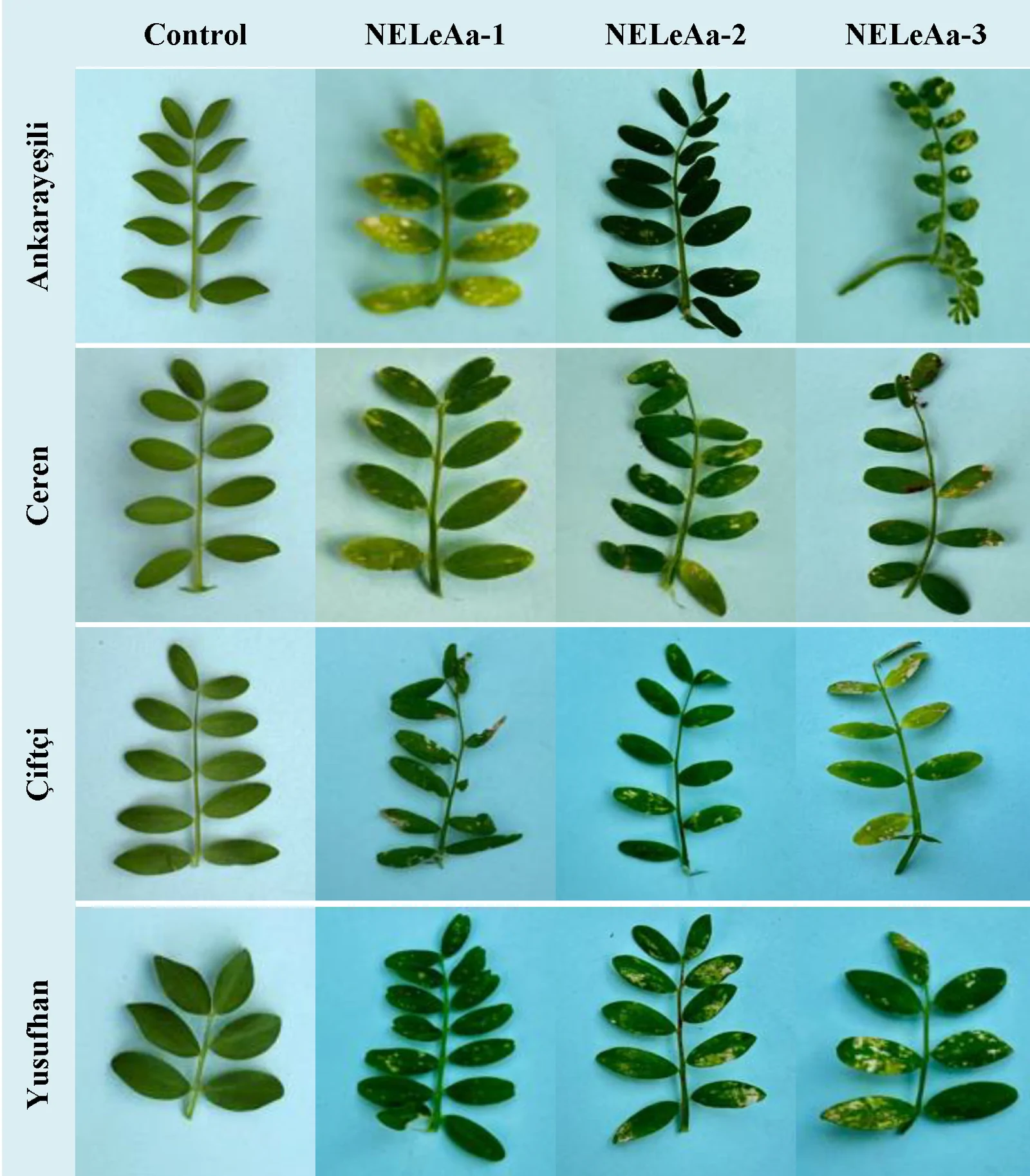
Pathogenicity symptoms caused by *Alternaria alternata* isolates on detached leaves of winter lentil cultivars under controlled conditions. Symptoms caused by Alternaria alternata isolates on detached leaves of winter lentil cultivars under controlled conditions, 14 days post-inoculation in pathogenicity tests.

**Figure 4:**
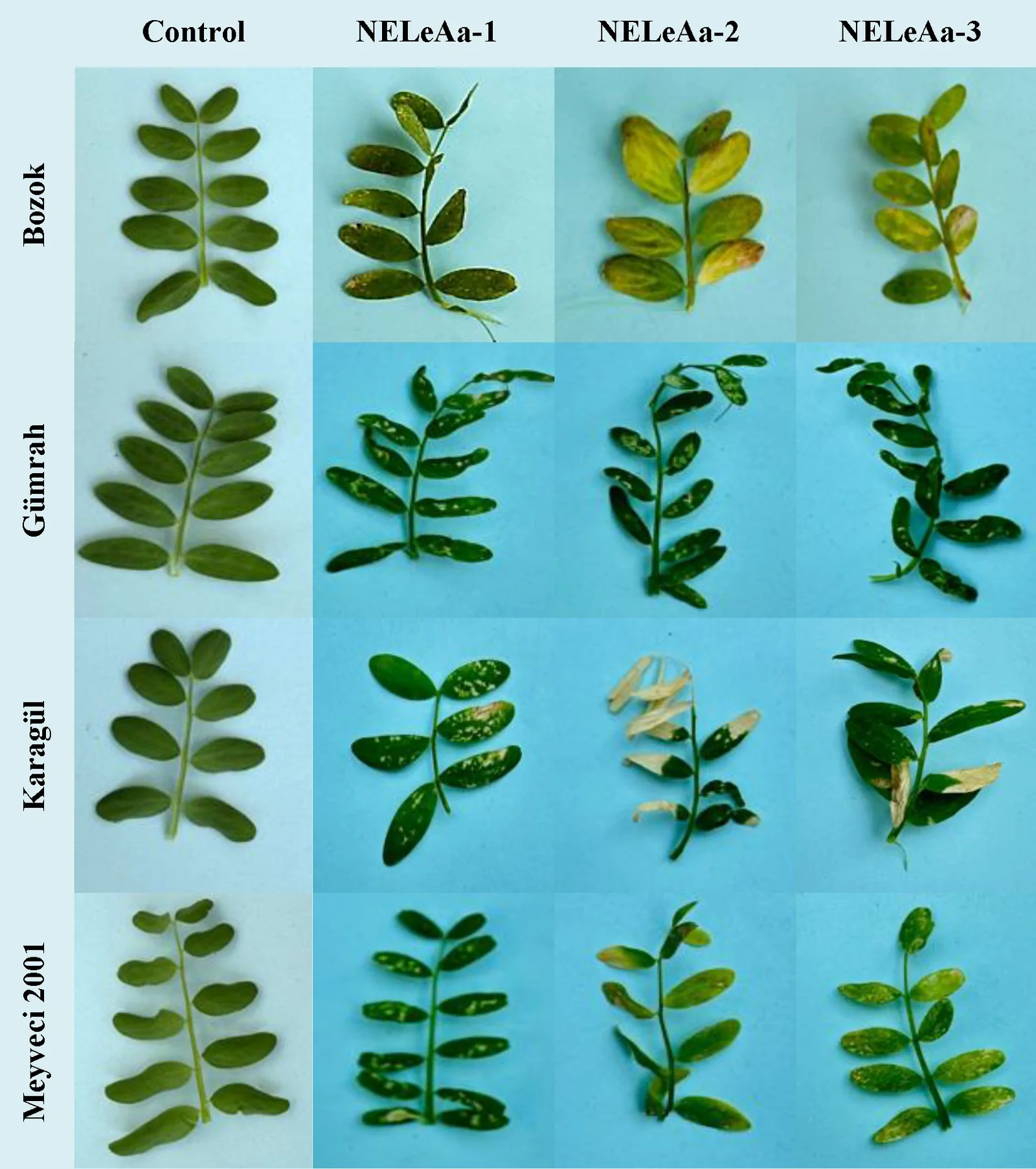
Pathogenicity symptoms caused by *Alternaria alternata* isolates on detached leaves of summer lentil cultivars under controlled conditions. Symptoms caused by Alternaria alternata isolates on detached leaves of summer lentil cultivars under controlled conditions, 14 days post-inoculation in pathogenicity tests.

The two-way ANOVA on the disease severity (DS) data showed significant effects for both ‘Cultivar’ and ‘Isolate’, as well as a significant ‘Cultivar × Isolate’ interaction (*P* < 0.001). As detailed in Table 4, DS percentages ranged from a minimum of 15.33% (NELeAa-1 on ‘Çiftçi’) to a maximum of 71.00% (NELeAa-2 on ‘Meyveci 2001’). Regarding the virulence of the pathogens, NELeAa-2 was the most aggressive isolate overall, particularly on summer cultivars. In contrast, NELeAa-1 showed a different virulence pattern, being more aggressive on the winter cultivar ‘Ankarayeşili’ (47.67%, MS) than NELeAa-2, which showed a lower severity (18.33%, MR). In terms of cultivar performance, a clear difference was observed between the two cultivation types. While winter cultivars such as ‘Ceren’ and ‘Çiftçi’ showed a consistent MR reaction across all isolates with DS values below 25%, summer cultivars were more susceptible. Specifically, ‘Meyveci 2001’ and ‘Karagül’ showed S reactions to the most virulent isolate (NELeAa-2), reaching DS scores of 71.00% and 53.67%, respectively.

**Table 4:** Disease severity (%) of different *Alternaria alternata* isolates on winter and summer lentil (*Lens culinaris* Medik.) cultivars.

**Cultivar Type**	**Cultivar**	**NELeAa-1** **(%)**	RC^*^	**NELeAa-2** **(%)**	RC^*^	**NELeAa-3** **(%)**	RC^*^
Winter	Ankarayeşili	47.67 ± 2.52 a	MS	18.33 ± 1.53 d	MR	31.67 ± 4.16 cd	MS
	Ceren	17.67 ± 2.52 c	MR	25.00 ± 3.00 cd	MR	23.33 ± 3.51 d	MR
	Çiftçi	15.33 ± 3.06 c	MR	22.33 ± 2.52 d	MR	20.67 ± 3.06 d	MR
	Yusufhan	37.00 ± 4.58 b	MS	40.33 ± 3.51 bc	MS	43.00 ± 2.65 b	MS
Summer	Bozok	31.67 ± 3.51 bc	MS	62.67 ± 2.52 a	S	59.33 ± 1.15 a	S
	Gümrah	30.67 ± 2.08 bc	MS	46.00 ± 2.65 b	MS	44.00 ± 1.00 b	MS
	Karagül	40.33 ± 2.08 ab	MS	68.00 ± 3.00 a	S	63.67 ± 3.51 a	S
	Meyveci 2001	46.67 ± 2.08 a	MS	71.00 ± 3.61 a	S	67.00 ± 3.00 a	S

**Notes.**

* Reaction code (RC): Assigned based on the mean infection area percentage according to the scale in Table 2: moderately resistant (MR), 11–25%, moderately susceptible (MS), 26–50%, and Susceptible (S), 51–75%. Values are expressed as mean percentage of infection area ± standard deviation (SD) of three replicates. Means within the same column followed by the same letter are not significantly different (*P* < 0.05) according to Duncan’s Multiple Range Test. Two-way ANOVA (Isolate x Cultivar): *F* = 28.7; *P* <  0.0001.

## Discussion

This study provides a characterization of *Alternaria alternata* as a significant foliar pathogen of lentil in Türkiye by integrating morphological, molecular, and pathological data. Our findings show the high pathogenic potential of *A. alternata*, especially on summer-type cultivars, and identify winter-type genotypes as potential sources of genetic resistance.

The morphology of our isolates is consistent with the taxonomic criteria for *A. alternata* as described by Rotem (1994) and Simmons (2007). The integration of morphological characteristics with molecular data is essential for accurate species identification within the *Alternaria* complex, helping to resolve taxonomic uncertainty (Wenneker et al., 2018). The classification of fungi such as *Alternaria*, whose sexual reproduction stage is unknown, is based on anamorphic features. In this case, since the classification is based entirely on morphological observation, the probability of error increases (Rotem, 1994). To prevent these inaccuracies, diagnostic criteria including conidia dimensions, number of septa, beak structure, and the number of spores in the conidial chain must be evaluated (Simmons, 1999; Rotem, 1994). Furthermore, morphological measurements should be supported by molecular identification techniques to ensure species differentiation (Cooke et al., 1998). In the molecular analysis, the use of the species-specific AaltFor/AaltRev primer set (Kordalewska et al., 2015) resolved the identity of the isolates, distinguishing them from closely related species within the *Alternaria* genus, such as *A. tenuissima* and *A. arborescens*. Phylogenetic analysis based on ITS sequences remains a fundamental tool for resolving the taxonomic positions of small-spored *Alternaria* species, as demonstrated in various recent studies (Milošević et al., 2022). This distinction is critical, as recent studies in regions like Iran and Algeria have reported a diverse spectrum of *Alternaria* species on lentil, including *A. chlamydosporigena* and *A. cantlous* (Kouadri, Bekkar & Zaim, 2023a; Kouadri, Bekkar & Zaim, 2023b; Zangakani, Abrinbana & Pouralibaba, 2025). Compared to traditional methods, PCR-based techniques are more advantageous in terms of time and sensitivity for the identification and differentiation of *A. alternata* from closely related species (Konstantinova et al., 2002). Our molecular findings align with recent reports from India (Roy et al., 2021) and Pakistan (Sajjad et al., 2024), where *A. alternata* remains the predominant species associated with pre-flowering and seed-borne blights.

Variation in virulence among *A. alternata* isolates from different geographical locations is a common phenomenon, often reflecting the pathogen’s high level of environmental adaptation (Hussain et al., 2023). Pathogenicity assays showed significant variation in virulence among the isolates (Table 4). Specifically, isolate NELeAa-2 was the most aggressive, inducing DS as high as 71.00%. The higher virulence of isolate NELeAa-2 may be associated with differences in toxin production or other pathogenicity-related factors, although these mechanisms were not investigated in the present study. The high virulence of isolate NELeAa-2 and the necrotic lesions surrounded by chlorotic halos (Figs. 3 and 4) might be related to differences in toxin production or other virulence factors. However, these mechanisms were not investigated in this study. As noted by Simmons (1999) and Rotem (1994), *A. alternata* pathotypes can produce toxins that damage host cell membranes and cause rapid necrosis. Similar observations have been reported in other legumes; for instance, in faba bean, *Alternaria* species such as *A. tenuissima* utilize specialized metabolites to colonize leaf tissues (Rahman, Honda & Islam, 2002).

A key finding was the clear difference in resistance between winter and summer lentil cultivars. Winter-type cultivars, such as ‘Ceren’ and ‘Çiftçi’, consistently showed MR reactions. This resistance might be related to both physiological and structural defense mechanisms. For instance, Roy et al. (2025) reported that some lentil genotypes can increase enzyme activities (such as peroxidase and polyphenol oxidase) and antioxidant levels during *Alternaria* infection, which helps limit fungal growth. Physical traits, including thicker leaf cuticles or different stomatal patterns, could also act as structural barriers against fungal entry. Overall, these factors might allow winter cultivars to resist Alternaria blight better than summer-type varieties. Conversely, the high susceptibility of summer cultivars like ‘Meyveci 2001’ indicates a genetic susceptibility to this pathogen. These results suggest that traits found in winter-type varieties are essential for developing sustainable management strategies in the region.

Our study shows that *A. alternata* is an important fungal agent associated with lentil leaf blight in Southeast Anatolia. Historically, surveys in this region focused more on soil-borne pathogens like *Fusarium* (Aydın, Koç & Sağır, 2004; Sağır, 1985). However, changing climate conditions, such as irregular spring rains and higher humidity, may contribute to more frequent Alternaria outbreaks on leaves. As mentioned in the national technical instructions for integrated pest management in lentils (Ministry of Food, Agriculture and Livestock, 2011), early detection is important. The high disease levels observed in this study (Table 4) are similar to the findings of Pouralibaba, Zangakani & Abrinbana (2025), who reported that *Alternaria* and *Stemphylium* species can infect rainfed legumes together and increase damage. Since Southeast Anatolia is the most important lentil production area in Türkiye (TUIK, 2025; Ministry of Agriculture and Forestry, 2019), the presence of aggressive isolates like NELeAa-2 is a risk for both yield and seed quality. Additionally, infected seeds may increase the potential risk of mycotoxin contamination (such as alternariol), making this not only a crop health problem but also a potential food safety concern (Ostry, Skarkova & Ruprich, 2002; Sajjad et al., 2024).

## Conclusion

This study shows that *Alternaria alternata* is an important fungal agent associated with Alternaria blight of lentil in Türkiye. Morphological observations combined with molecular analyses enabled reliable identification of the isolates. Based on the pathogenicity tests, ‘Ceren’ and ‘Çiftçi’ were identified as the most resistant varieties among the tested materials. The summer-type cultivars evaluated in this study were generally more susceptible to the disease, the introgression of resistance traits from these winter-type varieties into summer backgrounds represents an essential strategy for future breeding. Improving lentil production in Türkiye may be linked to developing new varieties resistant to such fungal pathogens. Therefore, continued collection of regional isolates and identification of locally adapted resistant germplasm will be important for breeding durable resistance against Alternaria blight in lentil.

## Supplemental Information

10.7717/peerj.21834/supp-1 Supplemental Information 1 Raw disease severity percentages for the 3 *Alternaria alternata* isolates as recorded during the pathogenicity tests *Alternaria alternata* isolates as recorded during the pathogenicity tests.

10.7717/peerj.21834/supp-2 Supplemental Information 2 Translation codebook

## Additional Information and Declarations

### Data Availability

The following information was supplied regarding data availability: The data is available in the Supplementary File.
