# Supplementary material for: Morphological and molecular characterization of Alternaria alternata causing Alternaria blight in lentil (Lens culinaris Medik.) and evaluation of the disease reactions of selected lentil cultivars in Türkiye
Source: PeerJ. 2026 Nov 9;14:e21834. doi: 10.7717/peerj.21834 (PMC13757733; doi:10.7717/peerj.21834)
Supplement: Supplementary file 1 [file peerj-14-21834-s002.docx]

| **Column / Term in Raw Data** | **English Translation / Description** |
| --- | --- |
| Cultivar Type | The growing season category of the lentil cultivar (Winter / Summer). |
| Cultivar | The name of the specific lentil cultivar used in the experiment. |
| Replications | The repetition number of the experiment for each cultivar (1, 2, or 3). |
| Control | The negative control group baseline value (0). |
| NELeAa-1 (Ankara) | Disease reaction / severity scores of the *Alternaria alternata* isolate sourced from Ankara. |
| NELeAa-2 (Konya) | Disease reaction / severity scores of the *Alternaria alternata* isolate sourced from Konya. |
| NELeAa-3 (Yozgat) | Disease reaction / severity scores of the *Alternaria alternata* isolate sourced from Yozgat. |
| Ankarayeşili | Ankarayesili (Lentil cultivar name written with English characters) |
| Çiftçi | Ciftci (Lentil cultivar name written with English characters) |
| Gümrah | Gumrah (Lentil cultivar name written with English characters) |
| Karagül | Karagul (Lentil cultivar name written with English characters) |
